# Osteoid osteoma of a metacarpal bone: A case report and review of the literature

**DOI:** 10.1186/1752-1947-2-285

**Published:** 2008-08-27

**Authors:** Efstathios Chronopoulos, Fragiskos N Xypnitos, Vassilios S Nikolaou, Nicolas Efstathopoulos, Dimitrios Korres

**Affiliations:** 1 2nd Orthopedic Department, Konstantopoulion Hospital, Athens University, Nea Ionia, Greece

## Abstract

**Introduction:**

Osteoid osteoma is a benign tumor of the growing skeleton. It presents with pain, which is usually worse at night. The radiographic features consist of a central oval or round nidus surrounded first by a radiolucent area followed by another area of sclerotic bone. In the hand, osteoid osteoma is more commonly located in the phalanges and carpal bones. The metacarpals are the least common sites for osteoid osteoma.

**Case presentation:**

We present a case of an osteoid osteoma of the left third metacarpal bone in a 36-year-old woman. The clinical and radiographic findings along with the surgical management of the lesion are presented. The pain disappeared immediately after the operation. At the 2-year follow-up, the patient was pain-free and there was no evidence of recurrence.

**Conclusion:**

Physicians should be aware of the unusual presence and the atypical clinical presentation of this benign lesion in the metacarpal bones of the hand.

## Introduction

Osteoid osteoma is a benign bone tumor of the growing skeleton representing approximately 10% of all benign bone neoplasias [1]. It usually affects children and young adults [1]. Heine in 1927 [2], Bergstrand in 1930 [3], and Jaffe in 1935 [4] identified osteoid osteoma as a clinical entity. Pain is often the only symptom of the disease and is typically described as mild and intermittent at first, becoming more constant and severe at night [5]. When the lesions appear in the hand, diagnosis is challenging for three reasons: first, the typical pain pattern may be absent; second, lesions in the hand may have unusual clinical signs and radiographic presentations; and third, histologic features may differ from classic osteoid osteomas, which occur in the long bones [6]. The metacarpals in particular are not a common site for osteoid osteoma and the diagnosis is often missed in the initial examination. We report a case of an osteoid osteoma in the third metacarpal, and describe the clinical presentation, radiological findings and successful outcome after surgical excision of the lesion.

## Case presentation

A 36-year-old woman was referred to our clinic in May 2005 with a 1-year history of pain in her left hand. The pain was constant but increased at night and after manual labor, and was reduced by non-steroidal anti-inflammatory agents. There was no history of injury.

There was a tender swelling of the head of the third metacarpal bone in the dorsum of the left hand at physical examination. The range of motion was not limited and there were no sensory disturbances. The grip strength of the left hand was slightly reduced, mainly due to pain.

**Figure 1:**
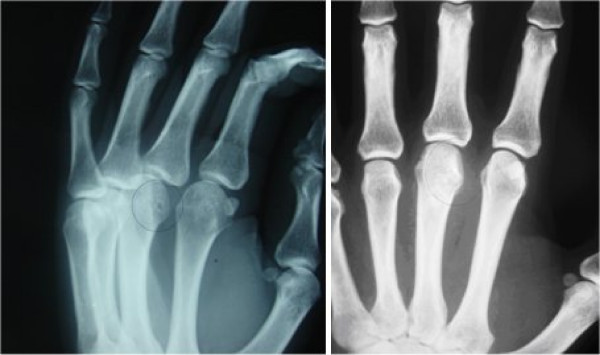
**Plain radiography of the left hand**. A small, oval, radiolucent lesion partially surrounded by sclerotic bone (left). No signs of recurrence at the 2-year follow-up (right).

Blood count and biochemical profile were within the reference ranges. The radiograph showed an oval nidus surrounded by a radiolucent ring (Fig. 1).

**Figure 2:**
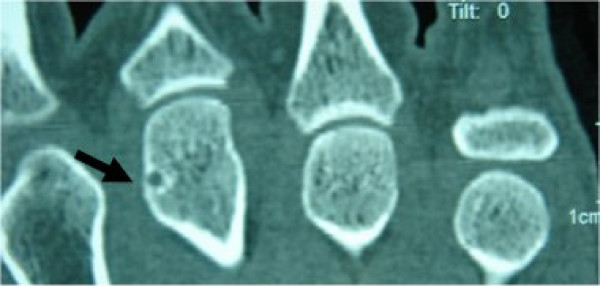
Computed tomography showing the radiolucent zone and the marked sclerosis around the lesion (arrow).

**Figure 3:**
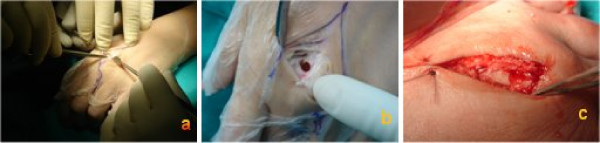
**Surgical procedure**. (a) Dorsal approach at the third metacarpal head. (b) Resection of the dorsal sclerotic bone. (c) The defect filled with an autogenous cancellous bone graft.

Computed tomography (CT) of the left hand clearly showed an oval radiolucent zone at the head of the third metacarpal bone and marked sclerosis around the lesion (Fig. 2). The history and clinical and radiographic findings pointed to the diagnosis of an osteoid osteoma of the head of the third metacarpal bone in the left hand. The patient was operated on 30 days later, by a dorsal approach (Fig. 3a), under a brachial plexus block. An en bloc excision of the nidus was performed using a small curette. A high-speed burr was also used to remove the sclerotic bone inside the lesion (Fig. 3b). The defect was filled with an autogenous cancellous bone graft (Fig. 3c). The hand was immobilized postoperatively with a splint.

Histological examination confirmed the diagnosis of osteoid osteoma. The pain disappeared immediately after the operation. At the 2-year follow-up, the patient was pain-free and there was no evidence of recurrence (Fig. 1).

## Discussion

Osteoid osteoma is a benign bone tumor of the growing skeleton representing approximately 10% of all benign bone neoplasias. It usually affects children and young adults. Normally the tumor does not exceed 1 cm in diameter [7]. The radiographic characteristic of osteoid osteoma is the central nidus, a 2 to 10 mm focus of osteoid nested in a more radiolucent fibrous stroma, surrounded by marginal sclerosis.

Osteoid osteoma usually occurs in the second and third decade of life. Male patients are more often affected than female patients by a ratio of 2:1, and the tumor is rare in the African-American population. It has a predilection for the lower extremity, with half or more of the lesions occurring in the femur and tibia, near the end of the shaft. Of the remaining lesions, approximately 30% are equally distributed among the spine, hand and foot [8].

Localization in the hand occurs with an incidence of only about 8% of all reported cases. Nevertheless, osteoid osteoma of the hand is well described in the literature. Allieu and Lussiez [9] and Ambrosia *et al*. [10] reported the largest series of hand osteoid osteomas. The phalanges are the most frequent sites for osteoid osteoma in the hand [11-13], followed by the carpal bones. The metacarpals are the least common sites for osteoid osteoma [14-16].

Trauma has been considered to be a contributing factor, although for others the correlation between injury and the onset of osteoid osteoma remains unclear [11]. Carroll [11] asserted that there is no direct correlation between them, but many cases have been reported in which an injury precedes the onset of the lesion. Kendrick and Evarts [17] reported that 15 out of their 36 cases had had an episode of initial trauma, and the incidence reported by Bednar *et al*. [18] was 11 out of 46 cases. Baron *et al*. [19] described 15 patients with post-traumatic osteoid osteoma. Uda *et al*. [14] reported a case of an osteoid osteoma of the metacarpal bone presenting after an injury.

Clinically, patients usually present with pain and swelling. The pain, which occurs in about 80% of patients, is more severe at night and is often relieved with salicylates or other non-steroidal anti-inflammatory agents that inhibit the production of prostaglandins by the lesion [20]. Several hypotheses have been proposed to explain the intensity of pain. Nerve endings might be stimulated by the high pressure owing to the increased blood flow within the tumor [21]. Nerve fibers, which are presumed to be components of the autonomic nervous system, are identified in the fibrous zone around the nidus [22]. Prostaglandins may directly stimulate free nerve endings inside or close to the tumor by lowering the nociceptive threshold [23]. A painless osteoid osteoma in a metacarpal has been reported by Basu *et al*. [15], nevertheless, all other metacarpal osteoid osteomas reported to date have presented with pain [7,9,10,12,13,23], as in our patient.

The diagnosis of an osteoid osteoma in the metacarpals may be difficult and is usually based on clinical and radiographic findings. Conventional radiographs can show the nidus as a small lytic spot surrounded by a radiolucent ring. However, about a quarter of osteoid osteomas are not detected on plain radiographs alone. In such cases, CT, bone scintigraphy, magnetic resonance imaging and angiography are useful in making the correct diagnosis [20]. Surgical treatment including excision of the nidus is usually curative [7], and is the treatment of choice. Recently, minimally invasive techniques, such as percutaneous trephine or drill resection [24,25], with or without the subsequent injection of ethanol [26,27] and thermal destruction with laser photocoagulation [28] or radiofrequency ablation [29], have been used for the removal or destruction of the nidus.

Recurrence of an osteoid osteoma is likely due to incomplete excision [30,31]. Usually, such recurrences have been recorded after curettage or drilling and rarely after an en bloc excision. Carroll [11] has stressed the need for careful radiological and microscopic control at the time of operation. Patients may experience a symptom-free interval after unsuccessful surgery. Recurrence of symptoms may indicate the presence of a second osteoid osteoma. Although such cases are rare, lesions with as many as three distinct nidi have been reported [32]. Most recurrences occur in the first 7 months after primary treatment [33] and have been associated with a nidus diameter of 1.0 to 1.5 cm [34].

## Conclusion

Osteoid osteomas of the hand are challenging to diagnose for several reasons. First, the typical pain pattern may be absent. Second, lesions in the hand may have unusual clinical signs and radiographic presentations. Third, histologic features may differ from classic osteoid osteomas, which occur in the long bones.

Osteoid osteomas of the metacarpal bones, although unusual, should be considered in the differential diagnosis of chronic pain in the hand of a young patient, presenting with or without a history of previous injury.

## Abbreviations

CT: Computed tomography.

## Competing interests

The authors declare that they have no competing interests.

## Authors' contributions

EC carried out the operation and conceived of the idea of presenting the case report. FNX assisted at the operation and in the preparation and drafting of the manuscript. VSN and NE assisted in the drafting of the manuscript. DK made the final check and approval of the submitted manuscript. All authors read and approved the final manuscript.

## Consent

Written informed consent was obtained from the patient for publication of this case report and any accompanying images. A copy of the written consent is available for review by the Editor-in-Chief of this journal.
